# Places where preschoolers are (in)active: an observational study on Latino preschoolers and their parents using objective measures

**DOI:** 10.1186/s12966-016-0355-0

**Published:** 2016-02-29

**Authors:** Ester Cerin, Tom Baranowski, Anthony Barnett, Nancy Butte, Sheryl Hughes, Rebecca E. Lee, Jason A. Mendoza, Debbe Thompson, Teresia Margareta O’Connor

**Affiliations:** Institute for Health and Ageing, Australian Catholic University, Melbourne, VIC 3000 Australia; School of Public Health, The University of Hong Kong, Hong Kong Hong Kong SAR, China; USDA/ARS Children’s Nutrition Research Center, Department of Pediatrics, Baylor College of Medicine, Houston, TX USA; Center for Health Promotion and Disease Prevention, College of Nursing and Health Innovation, Arizona State University, Phoenix, Arizona USA; Department of Pediatrics, University of Washington and the Seattle Children’s Research Institute, Seattle, WA USA

**Keywords:** Physical activity locations, Global positioning system, Accelerometry, Preschool-aged children, Parenting practices, Neighborhood perceptions, Sedentary behavior

## Abstract

**Background:**

To combat the disproportionately higher risk of childhood obesity in Latino preschool-aged children, multilevel interventions targeting physical (in) activity are needed. These require the identification of environmental and psychosocial determinants of physical (in) activity for this ethnic group. The objectives were to examine differences in objectively-measured physical activity and sedentary behavior across objectively-determined types of locations in Latino preschool-aged children; and determine whether the differences in physical activity by location were greater in children of parents with higher neighborhood-safety perceptions and physical activity-supportive parenting practices.

**Methods:**

An observational field study was conducted in Houston (Texas, USA) from August 2011 to April 2012. A purposive sample of Latino children aged 3–5 years and one of their parents (*n* = 84) were recruited from Census block groups in Houston (Texas) stratified by objectively-assessed high vs. low traffic and crime safety. Seventy-three children provided valid data. Time spent outdoors/indoors tagged with geographic locations was coded into location types based on objective data collected using Global Positioning Systems units that children wore >8 hr/day for a week. Physical activity parenting practices, perceived neighborhood-safety, and demographics were reported by parents. Time spent in sedentary behavior and moderate-to-vigorous physical activity was measured based on objective data collected using accelerometers (motion sensors) that children wore >8 hr/day for a week.

**Results:**

The odds of children engaging in moderate-to-vigorous physical activity were 43 % higher when outdoors than indoors (95 % confidence interval: 1.30, 1.58), and the odds of being sedentary were 14 % lower when outdoors compared to indoors (95 % confidence intervals: 0.81, 0.91). This difference depended on parental neighborhood-safety perceptions and parenting practices. Children were most active in parks/playgrounds (30 % of the time spent in moderate-to-vigorous physical activity) and least active in childcare/school settings (8 % of the time spent in moderate-to-vigorous physical activity).

**Conclusions:**

Objectively-assessed time spent in specific locations is correlated with physical activity and sedentary behavior in Latino preschoolers. Interventions and policies should identify ways to engage Latino preschool-aged children in more physical activity and less sedentary behavior while in childcare, and encourage parents to spend more time with their young children in parks/playgrounds and other safe outdoor places.

**Electronic supplementary material:**

The online version of this article (doi:10.1186/s12966-016-0355-0) contains supplementary material, which is available to authorized users.

## Background

Latinos are the fasted growing population in the United States (US) [1]. Compared to other ethnicities, Latino preschool-aged children have a disproportionately higher risk of childhood obesity [2] and show worse profiles of biomarkers related to cardiovascular disease risk in association with overweight/obesity [3]. Initiatives for increasing physical activity (PA) and reducing sedentary behavior (SB) have been acknowledged as key obesity prevention strategies in early childhood [4]. This also applies to Latino preschool children who were found to be more likely to have a healthy body mass index (BMI) if engaging in more moderate-to-vigorous physical activity (MVPA) [5]. Moreover, a recent integrative review on the determinants of obesity in this population concluded that decreased PA in Latino preschool-age children was a consistent contributor to obesity, while the evidence for nutrition-related factors (maternal feeding practices and beliefs, food choices) of obesity was inconsistent and inconclusive [6].

Both physical and social environmental factors influence young children’s PA and SB [7–9]. Time spent outdoors, in parks or playgrounds was found to be one of the strongest predictors of children’s PA [10–12]. Yet, with the exception of a small pilot study [12], this evidence pertained to non-Latino school-aged children, and was based on subjective proxy reports of child’s location and/or activity, which are often inaccurate. Examining the locations where PA and SB occur specifically among Latinos is important because Latinos may relate to their neighborhood and surroundings differently compared to other ethnicities, emphasizing the need to understand the environmental context in which PA occurs among young Latino children. In fact, the current PA literature points to differences in exposure to environmental factors and associations of environment with PA between Latino and non-Latino children [13, 14]. For example, Latino preschool-aged children had less active-play equipment at home, spent less time outdoors, were exposed to more parental rules/restrictions for PA, and received less parental encouragement for PA than their Anglo-American counterparts [13]. Latino parents differed from the Anglo-American counterparts in the factors they used in selecting play spaces for their young children [15]. Ethnicity was also found to moderate the association between access to safe parks and engagement in regular PA [16].

The main aim of this study was therefore to estimate differences in accelerometry-assessed MVPA and SB between types of locations (e.g., home, childcare, park, outdoor, indoor) visited by a sample of Latino preschool-aged children residing in Harris County, Texas, a region with a large proportion of Hispanic or Latino population (~42 % in 2013). In line with previous research, we hypothesized that children would be more active and less sedentary in places associated with fewer physical or social restrictions for PA, i.e., outdoors than indoors [13]; in parks/playgrounds than at home; and at home than at commercial establishments (e.g., businesses, shops and restaurants) [12].

Given that parental behavior and attitudes largely determine young children’s opportunities for PA participation [17–20], a secondary aim of this study was to examine whether between-location differences in child’s PA and SB depended on PA-related parenting practices and parental perceptions of neighborhood safety (moderators of location-PA and/or location-SB associations). We hypothesized that between-location differences in PA and SB would be greater in children of parents with more positive neighborhood-safety perceptions and parenting practices supportive of PA. This is because such parents might allow their children to be more active in appropriate places than parents who have safety concerns or do not encourage participation in PA. Additionally, we explored the moderating effects of child gender and weight status on the above between-location differences in children’s PA and SB.

## Methods

### Participants and recruitment

Latino parents and their preschool-aged children (3–5 years old; one preschooler enrolled per family) were recruited from census block groups (Harris County, Texas) cross-stratified by objectively-assessed traffic and crime safety into four strata (low traffic/low crime; low traffic/high crime; high traffic/low crime; high traffic/high crime). The number of participants per stratum (in the order presented above) was 25, 16, 13, and 19, and represented 56 different census block groups in Harris County. Details about the traffic and crime safety indices used to cross-stratify block groups were previously described [18]. This sampling strategy was adopted to maximize the variability of environmental exposures (neighborhood characteristics) and because neighborhood safety may impact children’s PA and PA-related parental practices [18, 21]. Parents or primary caregivers (here forth called parents) were recruited via various channels (e.g., community organizations). Eligibility criteria were being Latino and the parent of a Latino 3–5 year-old child living in the Houston area. Ethnicity was determined by self-identification as per US Census procedures [22] using the commonly-asked question “Are you Hispanic or Latino?”. This question was asked twice: upon recruitment and during the study as part of a socio-demographic questionnaire. Exclusion criteria included: preschool children with a disease or disability preventing them from participating in PA, and parents who did not self-identify as Latino or were unable to read and write in English or Spanish. Eligible parents provided informed written consent (in Spanish or English) to participate in the study and allow their 3–5 year old child wear an accelerometer (PA monitor) and Global Positioning System (GPS) unit for a week. Data were collected from August 2011 to April 2012 (90 % during fall to spring; average high temperature range 62–95 °F; average low temperature range 41–74 °F). Participants received $50 in compensation for complying with the study protocol. The Baylor College of Medicine Institutional Review Board approved the study.

### Procedure

Research staff met the participating parent and child in their home. After providing signed consent for themselves and permission for their child to participate, the parent completed a demographic questionnaire on a hard copy form and the rest of the questionnaires (see Survey Measures below) on a Personal Digital Assistant, after being assigned a participant code. The questionnaires were available in English and Spanish. Research staff were bilingual and gave participants the option of completing the questionnaires in English or Spanish. Only the preferred language version was provided to the participating parents.

Children’s height (to the nearest 0.1 cm; without shoes) and weight (to the nearest 0.1 kg; without shoes and with light clothing) were measured twice by research staff. Children’s BMI (kg/m^2^) was calculated and the US Centers for Disease Control and Prevention growth chart data were used to determine age- and gender-specific BMI z-scores [23].

Children simultaneously wore QStarz BT100X GPS units (QStarz International Co., Taiwan) and Actigraph GT3X accelerometers (Actigraph, Pensacola, USA) for a week. The clocks of the monitors were synchronized to the Universal Time Clock. PA data were collected at 15-second epochs [24], while GPS data were collected at 30-second epochs. The accelerometer was worn on the right hip, and the GPS unit on the left hip. Parents were instructed to remove the monitors before the child went to bed at night or engaged in water activities. They could remove the GPS unit whenever they did not want their child’s location to be tracked. Parents documented in a standardized log when the child removed the monitors. Data documented as non-wear times and periods of 30+ minutes of recorded zero accelerometer counts were removed from analysis. Accelerometer data were considered ‘valid’ if there were ≥480 min of activity data/day for 4+ days, including one weekend day. Participants with invalid accelerometer data were asked to re-wear the monitors for additional days. Allowing for re-wears, 82 of the 84 children enrolled (96 %) had valid accelerometer data, and 73 of them also had valid (non-missing) GPS data on locations visited (geographical coordinates). Sixty-six of these 73 children also had valid GPS data on whether they were *indoors*, *outdoors,* or *in vehicle* (see below). The socio-demographic characteristics of these two groups (73 and 66 children) are reported in Table 1. No substantial differences in socio-demographics (child’s age and gender; parental age, gender, educational attainment, employment status, household income, and language spoken at home) were found between participants with valid vs. non-valid GPS data (|Cohen’s d| <0.20).

**Table 1:** Parent and Child Socio-Demographics, Physical Activity (PA)-Related Parenting Practices, and Parental Perceived Neighborhood Safety

Variables	Whole sample (*n* = 73)	Sub-sample with GPS- estimated outdoor/indoor time (*n* = 66)
Parent characteristics ears (mean, SD)	32.3 (6.1)	31.5 (5.6)
Age, mean (SD)	32.6 (6.8)	32.9 (6.8)
Born in the US, n (%)	30 (41 %)	27 (41 %)
Country of origin, n (%)		
Mexico	54 (74 %)	49 (74 %)
El Salvador	9 (12 %)	8 (12 %)
Other Latin American countries	10 (14 %)	9 (14 %)
Education, n (%)		
< High School	19 (26 %)	17 (26 %)
High School/GED	28 (39 %)	26 (39 %)
> High School	25 (34 %)	23 (35 %)
Not answered	1 (1 %)	NA
Current employment status, n (%)		
Not employed	45 (62 %)	42 (64 %)
Employed	27 (37 %)	24 (36 %)
Not answered	1 (1 %)	n/a
Total household income, n (%)		
≤ $19 k	25 (34 %)	23 (35 %)
$20 k–$49 K	32 (44 %)	29 (44 %)
≥ $50 k	12 (16 %)	10 (15 %)
Unknown/No answer	4 (6 %)	2 (6 %)
Primary language spoken in household, n (%)		
English	9 (12 %)	8 (12 %)
Spanish	35 (48 %)	32 (48 %)
Both	29 (40 %)	26 (40 %)
Child characteristics		
Age in years, mean (SD)	4.5 (0.8)	4.5 (0.8)
Gender, n (%) Male	42 (58 %)	38 (58 %)
Weight status, n (%)		
Normal weight	35 (48 %)	32 (48 %)
Overweight	15 (21 %)	13 (20 %)
Obese	23 (31 %)	21 (32 %)
Hours/week spent in childcare center, n (%)		
None	45 (62 %)	40 (60 %)
Up to 10 hours	5 (7 %)	4 (6 %)
11–20 hours	9 (12 %)	9 (14 %)
21+ hours	14 (19 %)	13 (20 %)
PA parental practices^a^, mean (SD)		
Parental engagement and structure	3.52 (0.56)	3.53 (0.55)
Promote screen time	2.41 (0.79)	2.40 (0.79)
Promote inactivity	1.71 (0.56)	1.69 (0.56)
Psychological control	1.90 (0.59)	1.90 (0.62)
Restriction for safety concern	2.74 (1.01)	2.77 (1.01)
Perceived neighborhood safety, mean (SD)		
Traffic safety^b^	2.94 (0.74)	2.94 (0.71)
Traffic hazards^b^	2.73 (0.86)	2.79 (0.84)
Stranger danger^b^	2.51 (1.10)	2.53 (1.08)
Signs of physical and social disorder^a^	2.17 (0.86)	2.22 (0.87)

*Notes:* All children were born in the US and participating parents were mothers; *GED* general educational development, *SD* standard deviation. ^a^possible range: 1–5. ^b^possible range: 1–4

### Measures

#### Survey measures

Parents completed a survey (in Spanish or English) validated in a large sample of Latino parents of preschool-aged children (*n* = 240) including the participants of this study [17]. The survey encompassed a socio-demographic questionnaire, PA parenting practices, and perceived aspects of neighborhood safety (signs of physical and social disorder; traffic safety and hazards; and stranger danger). *PA parenting practices* were assessed using the Preschooler’s Physical Activity Parenting Practices instrument [17] with five subscales: one measuring parenting practices encouraging child PA (15 items), the others measuring parenting practices that discourage child PA [promote inactive transport (three items), promote screen time (three items), psychological control (manipulation of child’s behavior to satisfy the parents’ needs; four items), and restriction for safety concerns (four items)]. This instrument had good factorial validity and test-retest reliability (ICCs: 0.56–0.85) [17]. *Perceived signs of physical and social disorder* were assessed using a modified version of the ‘Disorder’ sub-scale from the Neighborhood Context scale [25] with 16 items rated on a 5-point scale [18]. *Perceived traffic safety* (3 items), *traffic hazards* (four items), and *stranger danger* (four items) were measured using an adaptation of the Neighborhood Environment Walkability Scale for Youth (NEWS-Y) with items rated on a 4-point Likert scale [18, 26]. The scales had moderate-to-excellent test-retest reliability [18]. The mean ratings on the items of each scale were used for the analyses.

#### Accelerometer and global positioning system data

Accelerometer data were processed using Pate’s cut points for preschool children [27] to determine sedentary time and MVPA, whereby <76 and ≥840 accelerometer counts per 30-second epochs were respectively classified as sedentary time and MVPA. Additionally, accelerometer counts collapsed to 30-second epochs provided a measure of “overall PA”. Accelerometer data were merged to GPS data.

Although full-day PA guidelines for preschool aged children have not been published in the US, and other countries’ PA recommendations vary as to their PA intensity recommendations for preschool-aged children [28], we used MVPA as an outcome because prior studies have associated MVPA rather than total light-moderate-vigorous PA (LMVPA) with a lower likelihood of overweight/obesity in Latino preschool-aged children [5, 29], and Latino preschool-aged children have higher prevalence of overweight/obesity and worse CVD-risk biomarker profiles associated with overweight/obesity than other US ethnic groups [6]. Additionally, wear time-adjusted accelerometer-assessed sedentary time represents the other side of the coin of wear time-adjusted accelerometer-assessed LMVPA, as the accelerometer count cut-point for SB is also the cut-point for LMVPA. Thus, in this study, all the findings pertaining to SB apply to LMVPA but in the opposite direction (i.e., a negative association between time spent outdoors and SB indicates a positive association between time spent outdoors and LMVPA).

Data from both monitors were processed using the Personal Activity Location Measurement System, (PALMS) version 1.4.0 (https://ucsd-palms-project.wikispaces.com/) [30, 31]. PALMS is an encrypted web application that simultaneously processes time-stamped accelerometer and GPS data to clean, filter, and detect locations and trips based on study specified settings and established algorithms. Locations where participants spent 3+ minutes were identified in PALMS for GPS coordinates falling within a 30 m buffer [31]. The locations identified in PALMS were viewed using Google Earth and coded into the following eight categories of destinations: home, other locations in apartment complex, other residential home, childcare/school/daycare, park/playground, any business/service without outdoor play area, any business/service with outdoor play area, and outside Houston. Multiple location points within a 50 m buffer from each participant’s home, other residential homes, or business/services without outdoor play area, were coded as one location [32]. Multiple location points within a 100 m buffer from each childcare center, school or day-care center were also treated as a single location [32]. Twenty percent of the participants’ processed data were coded for location by two independent coders with a corresponding Kappa of 0.85 (95 % CI: 0.81–0.89) indicating excellent agreement.

PALMS also identified participants’ trips between locations. Mode of travel differentiated vehicular vs. non-vehicular trips, with motorized vehicle speed cut-off of 25 km/h as per PALMS previously validated default settings [33]. Indoor, outdoor and in-vehicle location points were also identified using PALMS validated algorithms [34], which we found to have good sensitivity (82 %) and specificity (77 %) in Houston.

### Data analyses

Descriptive statistics were computed for socio-demographic characteristics, moderator variables (e.g., parental perceived neighborhood safety), participant-aggregated PA and SB variables, and participant-aggregated contextual (location) variables. Participant-aggregated variables were computed to provide an overview of children’s daily average levels of PA and SB and time spent in various types of locations. Participant-aggregated PA and SB variables included: average min/day of SB and MVPA; average accelerometer counts per 30 s; percentage of accelerometer wear time spent sedentary and in MVPA. Participant-aggregated contextual variables included the percentage of valid accelerometer/GPS time spent outdoors, indoors, in vehicle, in transit, and at eight types of locations. We computed percentage time spent in childcare/school/daycare for all participants as well as for only those whose parents reported they were enrolled in childcare/school/daycare. To examine whether time spent in different locations and average daily PA and SB differed by child’s gender and weight status, generalized linear models with robust standard errors accounting for census block group clustering [35].

#### Associations of contextual variables with physical activity and sedentary behavior

Thirty-second epoch-level rather than participant-aggregated data were used to examine associations of contextual variables with PA and SB. Epoch-level PA and SB variables included accelerometry counts per 30 s, engaging in SB in a specific 30 s epoch (no = 0; yes = 1), and engaging in MVPA in a specific 30 s epoch (no = 0; yes = 1). Epoch-level location (exposure) variables encompassed two categorical variables: indoor/outdoor/in vehicle (indoor = reference category), and location type (home = reference category).

Given the large amount of data and the need to account for temporal and spatial autocorrelation [36], regression models of PA and SB could not be performed simultaneously on all participants, but were run for each participant separately. This yielded two regression models (one with outdoor/indoor/in vehicle as the predictor, the other with eight location types as the predictor) per participant per outcome (MVPA, SB and accelerometer counts per 30 s), for a total of six regression models per child. Generalized additive mixed models (GAMMs) [37] were the type of regression models used for this purpose. GAMMs were used because they can model counts (accelerometer counts per 30 s epochs) as well as binary variables (engaging or not engaging in MVPA and SB) while accounting for spatial and temporal autocorrelation [37].

GAMMs of accelerometer counts per 30 s used a negative binomial distribution and a logarithmic link function (appropriate for positively skewed count variables). The other two PA outcomes were binary and were modelled using a binomial distribution and a logit link function. All GAMMs accounted for dependency at the day level (the fact that PA and SB may vary across different days). To account for correlated errors at successive within-day time points a correlation structure with a continuous autoregressive process of order 1 was used [37]. Residual spatial correlation was accounted for by including in the GAMMs a non-parametric smooth interaction term of the geographical coordinates (longitude and latitude) corresponding to participants’ locations at specific 30-second epochs. GAMMs were conducted in R using the package ‘mgcv’ [37]. Adjustment for spatial and temporal residual correlation was necessary to obtain valid estimates of regression coefficients and standard errors [36–38].

After obtaining participant-specific estimates of associations of the two contextual variables with PA and SB using the GAMMs described above, it was necessary to synthesize the findings for the whole sample. To achieve this, the participant-specific regression coefficients and standard errors variance-covariance matrices obtained from the above GAMMs were entered in meta-regression models (i.e., a meta-analysis with predictor variables). These meta-regression models treated the sample of participants as a random sample of ‘studies’ (each participant representing one study) with multiple correlated findings [39]. ‘A finding’ corresponded to the difference in a participant’s mean outcome (e.g., accelerometer counts per 30 s) between the reference category for a location (e.g., indoors) and another type of location (non-reference e.g., outdoors). Two sets of meta-regression models were constructed, one for each of the two categorical contextual variables used in this study (indoor/outdoor/in vehicle; types of locations). These meta-regression models provided estimates of the average associations between contextual and PA/SB variables for the whole sample. Meta-regression models of indoor vs. outdoor time also examined child gender, child weight status, PA-related parenting practices, and parental perceptions of neighborhood safety as potential moderators of the associations between being indoors/outdoors and the PA/SB outcomes. This was done by entering the moderators as predictors in the meta-regression models. The moderating effects of these variables on the associations between location types and PA/SB outcomes were not examined because the number of children visiting location types other than home was small (7 to 71 children; Table 2). Statistically significant moderators were investigated by estimating the associations of contextual and PA/SB variables at three different values of the moderator (mean and ±1 standard deviation from the mean). Meta-regressions were conducted in R using the procedures outlined by Hox [39] and the package ‘metafor’ [40]. A probability level of 0.05 was adopted.

**Table 2:** Descriptive Statistics of Physical Activity, Sedentary Behavior and Contextual Variables

Variables	Whole sample (*n* = 73)	Sub-sample with GPS- estimated outdoor/indoor time (*n* = 66)
Accelerometry/GPS data validity		
Accelerometer wear time, min/day (SD)	702 (66)	702 (67)
Valid days of accelerometer wear (SD)	6.5 (1.3)	6.4 (1.2)
Accelerometer/GPS valid data, min/day (SD)	644 (85)	647 (86)
Valid days of accelerometer/GPS data (SD)	6.4 (1.2)	6.4 (1.2)
Physical activity variables		
Sedentary, min/day (SD)	371 (70)	371 (69)
MVPA, min/day (SD)	84 (40)	86 (41)
Counts per 30 seconds (SD)	313 (119)	320 (123)
% time sedentary^a^	53 (8)	53 (8)
% time in in MVPA^a^	12 (5)	12 (5)
Contextual variables		
% time spent outdoors/indoors/in vehicle (SD)^b^		
Outdoors	-	35 (20)
Indoors	-	59 (22)
In vehicle	-	6 (5)
	Whole sample (*n* = 73)	In those visiting a location [n]^c^
% time at specific locations (SD)^b^		
No fixed location (in transit/trips)	12 (7)	12 (7) [73]
Home	57 (22)	57 (22) [73]
Other locations in apartment complex	2 (5)	4 (7) [29]
Other residential home	7 (12)	9 (13) [58]
Child-care/school/daycare	14 (14)	22 (15) [45]
		30 (10) [28]^d^
Park/playground	1 (2)	4 (5) [13]
Other (any business/service) without outdoor play area	5 (5)	5 (5) [71]
Other (any business/service) with outdoor play area	1 (5)	3 (7) [39]
Outside Houston	1 (3)	8 (9) [7]

*Notes:* ^a^% time refers to % of valid accelerometry time. ^b^% time refers to % of valid accelerometry/GPS unit time. ^c^Number of children who visited specific locations within the study period varied and is reported in square brackets [n]; ^d^ In children whose parents reported they were enrolled in child-care/school/daycare. *GPS* global positioning system, *MVPA* moderate-to-vigorous physical activity, *SD* standard deviation

## Results

Table 2 shows the descriptive statistics of all PA and contextual variables. Participating children spent, on average, 84 (SD = 40) minutes per day in MVPA, corresponding to 12 % of the time they wore the accelerometers (>10 h/day). They spent 53 % of the remaining overall monitor-wear time in sedentary activities. Home was the location children spent, on average, most of their monitor-wear time (57 %), followed by childcare/school/daycare (30 % for children enrolled in childcare; 22 % for children visiting such a facility during the study; 14 % for the whole sample, which includes those that never visited childcare setting), and other residential locations. Only 18 % of the children spent time in a park during the study week, which represented 4 % of their total monitor-wear time. No differences between weight-status categories were observed in the examined variables. Only between-gender differences in time spent in locations in the apartment complex other than home and outside Houston were found (Additional file 1). In children who had valid outdoor/indoor GPS data, 59 % of valid GPS/accelerometer wear time was spent indoors (~6.5 out of 10.5 h) and 35 % outdoors (~3.5 h). Locations that were associated with a larger proportion of outdoor time were parks/playgrounds (88 %) followed by being out of town (78 %), while childcare/school/daycare were the locations associated with the lowest percentage of outdoor time in children enrolled (13 %) and not enrolled (14 %) in childcare/school/daycare (Table 3) [Note:, a few children whose parents had reported on the demographic survey they did not attend childcare/school/daycare, were found to spend time in these types of locations during the study period according to GPS data].

**Table 3:** Percentage of Time Spent at Specific Locations by Time Spent Outdoors/Indoors/In Vehicle (*n* = 66)

Location		Outdoors	Indoors	In vehicle
	n	Mean (SD)	Mean (SD)	Mean (SD)
No fixed location (in transit/trips)	66	12 (14)	4 (5)	84 (15)
Home	66	36 (18)	64 (24)	0 (0)
Other locations in apartment complex	23	39 (20)	58 (22)	3 (3)
Other residential home	52	36 (20)	63 (21)	1 (1)
Child-care/school/daycare	41	14 (13)	85 (12)	1 (1)
	26^a^	13 (14)	86 (17)	1 (1)
Park/playground	13	88 (10)	10 (9)	2 (2)
Other (any business/service) without outdoor play area	64	40 (20)	56 (25)	4 (3)
Other (any business/service) with outdoor play area	34	28 (21)	70 (21)	2 (1)
Outside Houston	5	78 (10)	19 (11)	3 (3)

*Notes. SD* standard deviation. *n* number of participants (children) that visited specific locations within the study period. ^a^In children whose parents reported they were enrolled in child-care/school/daycare

### Associations of contextual variables with physical activity and sedentary behavior

Children were most active outdoors and least active when in a vehicle. The latter findings serves as a validation for the PALMS web application which used GPS data to identify time spent walking, being stationary in a specific location, and in motorized trips (in a moving vehicle). The odds of children engaging in MVPA were 43 % higher when outdoors than indoors, and the odds of being sedentary were 14 % lower when outdoors, compared to indoors (Table 4). When compared to home, where children spent most of their time, being in other locations in the apartment complex and at a park/playground was associated with higher levels of PA and less sedentary time (Table 5). Being at a park/playground predicted 84 % higher average accelerometer counts than being at home, and the odds of engaging in MVPA were 341 % higher than when at home. Time spent at other residential homes were associated with lower odds of being sedentary but not with higher odds of MVPA. Being in any business or service was associated with lower levels of activity but not with lower odds of being sedentary. Being in a childcare center or school was associated with the lowest level of activity and the highest odds of being sedentary of all other locations (Table 5).

**Table 4:** Physical Activity and Sedentary Behavior Outcomes by Indoor, Outdoor and ‘In Vehicle’ Locations (*n* = 66)

Physical activity outcomes	Descriptive statistics – mean (SD)			Meta-regression analyses – average regression coefficient (95 % confidence intervals)^a^			
	Indoors	Outdoors	In vehicle	Indoors vs Outdoors	*p*	Indoors vs In vehicle	*p*
Accelerometer counts per 30 seconds	313 (525)	361 (594)	121 (239)	1.13 (1.09, 1.16)	<.001	0.56 (0.51, 0.62)	<.001
Engaging in sedentary behavior (% time)	46 (50)	43 (50)	64 (48)	0.86 (0.81, 0.91)	<.001	1.59 (1.38, 1.82)	<.001
Engaging in MVPA (% time)	11 (35)	14 (35)	2 (15)	1.43 (1.30, 1.58)	<.001	0.39 (0.34, 0.45)	<.001

*Note: MVPA* moderate-to-vigorous physical activity. % time refers to % of valid accelerometry/GPS time. *SD* standard deviation represents the total within- and between-person variability in the physical activity outcomes. *p* *p*-value. ^a^Average regression coefficients for accelerometer counts per 30 s represent the proportional difference between average accelerometer counts when indoors vs. outdoors, and when indoors vs. in vehicle. Average regression coefficients for sedentary behavior and MVPA represent the odds ratios of engaging in such activities when indoors vs outdoors and when indoors vs in vehicle. Individual regression models (that were included in the meta-regression) were adjusted for temporal and spatial autocorrelation

**Table 5:** Physical Activity and Sedentary Behavior Outcomes by Location Types (*n* = 73)

		Physical activity outcome					
Location type	n	Accelerometer counts per 30 seconds		Engaging in sedentary behavior (% time)		Engaging in MVPA (% time)	
Descriptive statistics		Mean (SD)		Mean (SD)		Mean (SD)	
Home	73	337 (560)		45 (50)		12 (33)	
Other locations in apartment complex	29	386 (472)		32 (47)		15 (35)	
Other residential home	58	361 (556)		41 (49)		13 (34)	
Child-care/school/daycare	45	247 (418)		51 (50)		8 (27)	
Child-care/school/daycare – in enrolled children only	28	227 (386)		51 (50)		7 (26)	
Park/playground	13	645 (716)		24 (43)		30 (46)	
Other (any business/service) without outdoor play area	71	276 (448)		47 (50)		9 (29)	
Other (any business/service) with outdoor play area	39	262 (441)		49 (50)		9 (29)	
Meta-regression analyses^a^		*e* ^*b*^ (95 % CI)	*p*	OR (95 % CI)	*p*	OR (95 % CI)	*p*
Home (reference category) vs …							
Other locations in apartment complex		1.25 (1.08, 1.45)	.003	0.67 (0.56, 0.80)	<.001	1.31 (1.08, 1.59)	.006
Other residential home		1.01 (0.88, 1.17)	.887	0.81 (0.69, 0.96)	.011	1.12 (0.95, 1.32)	
Child-care/school/daycare		0.64 (0.56, 0.74)	<.001	1.26 (1.07, 1.49)	.006	0.67 (0.57, 0.78)	<.001
Child-care/school/daycare – in enrolled children only		0.78 (0.70, 0.87)	<.001	1.23 (1.03, 1.54)	.022	0.66 (0.53, 0.83)	<.001
Park/playground		1.84 (1.48, 2.28)	<.001	0.37 (0.30, 0.47)	<.001	4.41 (3.28, 5.85)	<.001
Other (any business/service) without outdoor play area		0.83 (0.72, 0.97)	.010	1.02 (0.82, 1.14)	.859	0.83 (0.71, 0.99)	.019
Other (any business/service) with outdoor play area		0.78 (0.67, 0.91)	.001	1.04 (0.87, 1.25)	.746	1.00 (0.82, 1.22)	.999

*Notes:* Locations outside Houston and no fixed locations/trips excluded from the analyses; % time refers to % of valid accelerometry/GPS time; *n* number of participants (children) that visited specific locations during the monitoring period, *MVPA* moderate-to-vigorous physical activity, *GPS* global positioning system, *SD* standard deviation representing the total within- and between-person variability in the physical activity outcomes, *OR* odds ratio, *95 % CI* 95 % confidence intervals, *e* ^*b*^ anti-logarithm of regression coefficient, *p p*-value. ^a^Antilogarithms of average regression coefficients (*e* ^*b*^) for accelerometer counts per 30 seconds represent the proportional difference between average accelerometer counts when home vs at other locations. Average regression coefficients for sedentary behavior and MVPA represent the odds ratios of engaging in such activities when home vs at other locations. Individual regression models (that were included in the meta-regression) were adjusted for temporal and spatial autocorrelation

### Moderators of associations

**Fig. 1:**
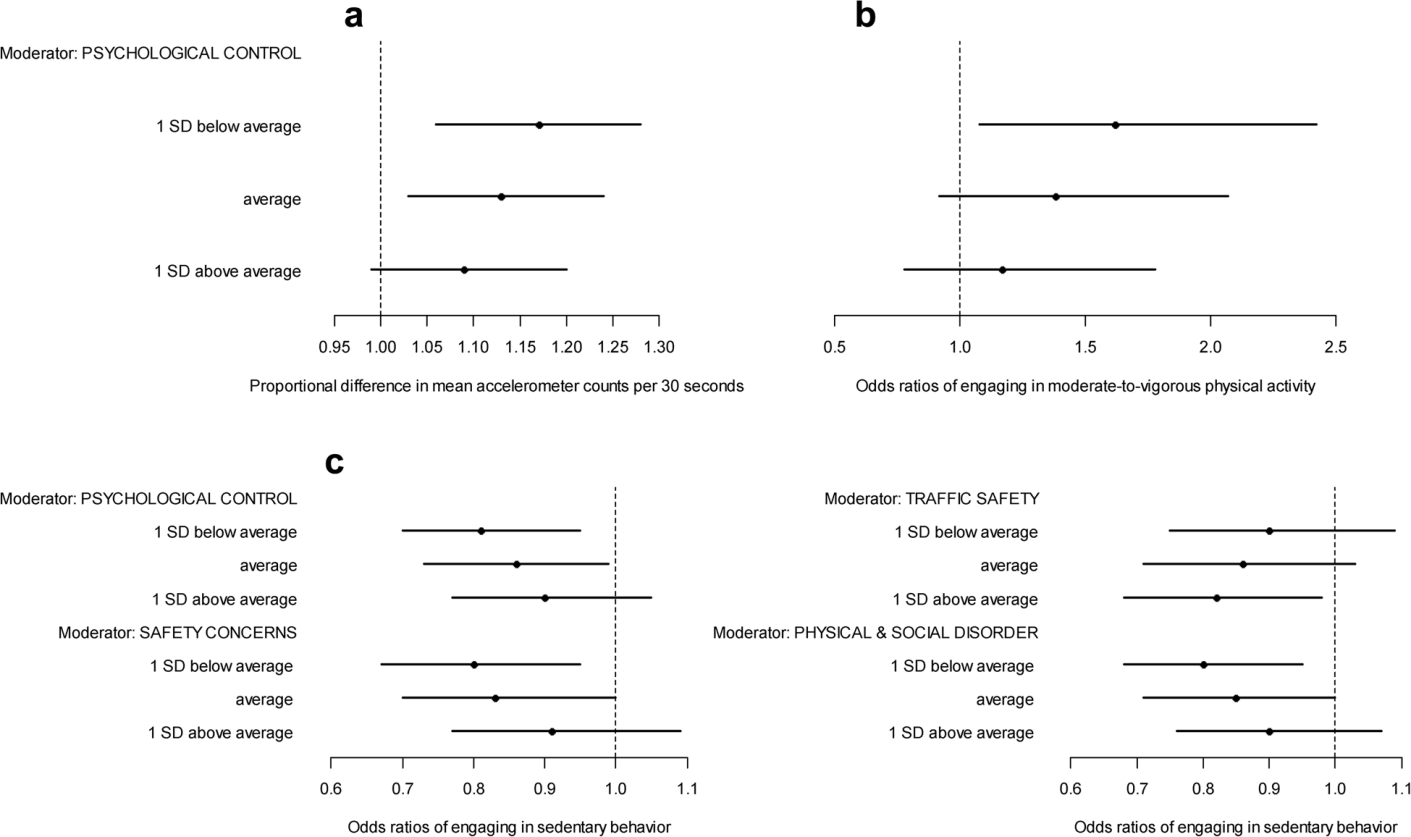
Moderators of Differences in Children’s Physical Activity and Sedentary Behavior between Indoor and Outdoor Locations. *Legend:* Differences are expressed as regression coefficients: proportional difference in mean accelerometer counts per 30 s (panel **a**); and odds ratios of engaging in moderate-to-vigorous physical activity (panel **b**) or sedentary behavior (panel **c**); lines are 95 % confidence intervals

Two PA-related parenting practices and two aspects of perceived neighborhood safety moderated the associations of PA outcomes with being outdoors vs. indoors (Fig. 1). Significant differences in accelerometry counts and odds of engaging in MVPA between indoor and outdoor locations were found only in children of parents who infrequently used psychological control as a practice to discourage child’s PA (Fig. 1, panels a and b). Significant indoor vs. outdoor differences in odds of being sedentary were found only in children of parents with average or lower scores on the parenting PA-discouraging practices of psychological control and restrictions for safety concerns, and on perceived signs of physical and social disorder, and above average scores on perceived traffic safety (Fig. 1, panel c).

## Discussion

In line with previous research in other populations [10, 11, 41–43], being in outdoor locations predicted children being more active than being in indoor locations. This was particularly the case for parks, playgrounds, and out-of-home locations within a residential complex. Yet, only one in five children visited a park or playground during the 7-day study period, and 40 % of children spent time in locations surrounding a residential complex for fewer than 30 min per day. Low proportions of daily PA located in parks and playgrounds have been previously reported in older children [44, 45], and have been attributed to poor park/playground accessibility and quality. As a recent study on preschoolers’ PA in childcare centers noted [43], PA interventions aiming to increase young children’s outdoor time at suitable and safe locations hold promise. These interventions may need to focus on enhancing the accessibility of safe outdoor places for play as well as increasing parents’ and preschool teachers’ awareness of the importance of spending more time with children outdoors [44, 45].

As previously observed, business/service locations were associated with lower levels of PA likely due to parents controlling their child’s behavior to a greater extent, or fewer opportunities for active play in such settings [17]. Yet, children were even less active in school and childcare settings, where enrolled children, on average, spent ~21 h/week, and more than half of that time was devoted to sedentary activities. In fact, schools and childcare centers were the places with the lowest level of PA, the highest level of SB, and the lowest percentage of outdoor time. While low levels of PA in preschool settings have been previously reported [42, 46–48], only one other recent study conducted in the United Kingdom (UK) has compared preschool-aged children’s PA and SB levels in school/childcare settings with other locations, albeit using parental reports to determine time spent in and out of childcare [49]. In the UK study, higher levels of activity were observed in childcare as compared to home. These have been attributed to the introduction of ‘free-flow’ policies allowing children in childcare to freely move indoors and outdoors for most of the day irrespective of weather conditions [49]. The low levels of PA in childcare observed in the present study are sobering given the increasing proportion of Latino preschoolers in childcare in the US (from 12 % in 2006 to 21 % in 2011) [50, 51], coupled with the number of hours many children spend in this environment (~29 h/week) [50]. Interventions and policies should identify ways to engage preschool children in more MVPA and less SB while in childcare [43].

As postulated by socio-ecological models [52], this study suggests that built and social environmental factors interact to shape Latino preschool-aged children’s PA and SB. Differences in PA and SB between outdoor and indoor locations depended on parental attitudes and practices related to neighborhood safety. These findings suggest that parents may restrict children’s spontaneous tendency to be more active outdoors if they perceive their neighborhood to be unsafe. It is thus important to provide access to objectively safe outdoor environments where parents can take their children to play and, at the same time, help parents with overprotective tendencies build more realistic and positive perceptions about the safety of these environments for their child to play. In a previous study on the same sample, we found that parental psychological control (i.e., manipulation of child’s behavior to satisfy the parents’ needs) was positively related to children’s PA [18]. In this study, high levels of psychological control were associated with smaller differences in PA and SB between outdoor and indoor locations. Parents of children who are spontaneously more active may tend to use psychological control to restrict their level of activity in general, which would explain why their children had similar levels of PA and SB in different contexts.

According to recent Australian, Canadian, and UK PA guidelines for preschool-age children [53], our sample exceeded the daily recommendation of 180 min of PA (usually operationalized as LMVPA). Similar levels of PA have been reported in other recent investigations on Latino [54] and other young children [55]. It remains to be seen whether the associations observed in this study hold in less active subgroups of children. Given that leading an active lifestyle can only be beneficial to health and that PA levels decline as children approach adolescence [56], investigating environmental factors that promote PA in this population remains an important public health issue.

This is the first study to use accelerometer and GPS monitors to capture objective data on locations, PA, and SB over a 1-week period in Latino preschool-aged children’s habitual environment. It is the first study to adjust for both spatial and temporal autocorrelation in determining the potential effect of different contexts on children’s PA and SB, thus, providing very robust estimates of associations. Limitations are the use of a non-probability sampling; a relatively small sample size; the correlational nature of the study not allowing establishment of causality; the lack of information on the social context in the various types of locations visited by the child (i.e., the person(s) a child was with); and the potential misclassification of outdoor vs. indoor time. This study estimated that children spent on average ~3.7 h per day outdoors, which seems high. Yet, using the same method, Tandon and colleagues [34] found that preschoolers spent outdoors ~1 h of their childcare time. A diary-based study conducted in Baltimore indicated an average of ~3.1 h of outdoor time in inner city preschool-aged children [57], while in Canada only ~2 h were reported [58]. A better climate in this study site (Houston; fall to spring) than in the other studies may account for more time spent outdoors. With regards to the lack of information on the social context associated with various types of locations, it is important to note that the presence of specific people (e.g., siblings, friends, parents, teachers, etc.) may affect a child’s activity level and, thus, the associations of specific types of location with children’s MVPA and SB [59, 60]. Future studies will need to establish the extent to which the social context confounds or moderates the differences in MVPA and SB across different types of locations.

## Conclusions

Latino preschool-aged children, irrespective of gender and weight status, were most active outdoors, and while in parks or playgrounds; and least active in school/childcare settings Differences in levels of children’s activity between outdoor and indoor settings were smaller if parents had less favorable perceptions regarding the safety of their neighborhood environment, and used parenting practices discouraging children’s PA. Interventions and policies should identify ways to engage Latino preschool-aged children in more physical activity and less sedentary behavior while in childcare, and encourage parents to spend more time with their young children in parks/playgrounds and other safe outdoor places.

## Additional file

Additional file 1: Table S1. Descriptive Statistics of Physical Activity and Contextual Variables by Child’s Gender. (DOCX 21 kb)

## Abbreviations

BMI: body mass index
CVD: cardiovascular disease
GAMMs: generalized additive mixed models
GPS: global positioning system
ICC: intraclass correlation
LMVPA: light, moderate and vigorous physical activity
MVPA: moderate-to-vigorous physical activity
NEWS-Y: neighborhood environment walkability scale for youth
PA: physical activity
PALMS: personal activity location measurement system
SB: sedentary behavior
SD: standard deviation
UK: United Kingdom
US: United States
