# Supplementary material for: Places where preschoolers are (in)active: an observational study on Latino preschoolers and their parents using objective measures
Source: Int J Behav Nutr Phys Act. 2016 Feb 29;13:29. doi: 10.1186/s12966-016-0355-0 (PMC4772489; doi:10.1186/s12966-016-0355-0)
Supplement: Additional file 1: Table S1. — Descriptive Statistics of Physical Activity and Contextual Variables by Child’s Gender. (DOCX 21 kb) [file 12966_2016_355_MOESM1_ESM.docx]

**Supplementary Table 1. Descriptive Statistics of Physical Activity and Contextual Variables by Child’s Gender**

| **Variables** | **Whole sample** | | **Sub-sample with GPS- estimated outdoor/ indoor time** | |
| --- | --- | --- | --- | --- |
|  | **Boys (n=42)** | **Girls (n=31)** | **Boys (n=39)** | **Girls (n=27)** |
| **Accelerometry/GPS data validity** |  |  |  |  |
| Accelerometer wear time, min/day (SD) | 702 (74) | 702 (54) | 700 (74) | 703 (58) |
| Valid days of accelerometer wear | 6.4 (1.4) | 6.7 (1.2) | 6.4 (1.3) | 6.5 (1.0) |
| Accelerometer/GPS valid data, min/day (SD) | 638 (91) | 652 (76) | 644 (91) | 650 (80) |
| Valid days of accelerometer/GPS data | 6.3 (1.3) | 6.6 (1.1) | 6.4 (1.3) | 6.5 (1.0) |
| **Physical activity variables)** |  |  |  |  |
| Sedentary, min/day (SD) | 369 (80) | 374 (66) | 371 (75) | 371 (69) |
| MVPA, min/day (SD) | 83 (25) | 85 (54) | 84 (25) | 88 (57) |
| Counts per 30 seconds (SD) | 316 (77) | 310 (160) | 321 (79) | 320 (169) |
| % time sedentary^1^ | 52 (7) | 53 (8) | 52 (8) | 53 (9) |
| % time in in MVPA^1^ | 12 (3) | 12 (7) | 12 (4) | 12 (7) |
| **Contextual variables** |  |  |  |  |
| % time spent outdoors/indoors/in vehicle (SD)^2^ |  |  |  |  |
| Outdoors | - | - | 35 (21) | 34 (19) |
| Indoors | - | - | 59 (24) | 61 (20) |
| In vehicle | - | - | 6 (6) | 6 (3) |
|  | **Whole sample** | | **In those visiting a location** | |
|  | **Boys (n=42)** | **Girls (n=31)** | **Boys [n]^3^** | **Girls [n]^3^** |
| % time at specific locations (SD)^2^ |  |  |  |  |
| No fixed location (in transit / trips) | 12 (7) | 13 (6) | 12 (7) [42] | 13 (6) [31] |
| Home | 61 (23) | 53 (19) | 61 (23) [42] | 53 (19) [31] |
| Other locations in apartment complex | 2 (6) | 0 (1) | 5 (8) [19] | 1 (1) [10]^a^ |
| Other residential home | 4 (7) | 10 (16) | 5 (8) [31] | 12 (17) [27] |
| Childcare/school/daycare | 12 (14) | 16 (15) | 21 (16) [24]  30 (11) [15]* | 23 (14) [21]  31 (9) [13]* |
| Park/playground | 1 (3) | 0 (1) | 6 (7) [7] | 2 (2) [6] |
| Other (any business/service) without outdoor play area | 5 (4) | 6 (6) | 5 (4) [40] | 6 (6) [31] |
| Other (any business/service) with outdoor play area | 2 (6) | 2 (4) | 4 (8) [24] | 3 (5) [15] |
| Outside Houston | 1 (3) | 0 (1) | 13 (9) [4] | 2 (2) [3]^a^ |

*Notes:* ^1^ % time refers to % of valid accelerometry time. ^2^ % time refers to % of valid accelerometry/GPS unit time. ^3^ The number of participants (children) that visited specific locations within the study period varied and is reported in square brackets [n]. * In children whose parents reported that they were enrolled in childcare/school/daycare. ^a^ p< .05 (significant difference between boys and girls). GPS = Global Positioning System; MVPA = moderate-to-vigorous physical activity; SD = standard deviation.
